# Society Meetings

**Published:** 1902-02

**Authors:** 


### SOCIETY MEETINGS.

The Niagara Falls Academy of Medicine held its regular monthly meeting at the office of Dr. C. E. McCarty, Monday evening, January 6, 1902. Dr. Herman Mynter, of Buffalo, read a paper, entitled, Total extirpation of prostate gland. Dr. N. W. Price read a paper on Indigestion.

THE Lake Erie Medical Society held its last regular meeting at Hotel Eggleston, Dayton, N.Y., Friday, January 3, 1902. The officers were· President, F. C. Beals; vice-president, C. H. Richardson; secretary-treasurer, A. L. Borden.

The program was as follows: Typhoid fever in military camps, A. H. Briggs, Buffalo; Military hospitals, J. G. Thompson; Gonorrhea, J. Henry Dowd, Buffalo; paper, James Taggert.

---

THE Buffalo Academy of Medicine held meetings during the month of January, 1902, as follows:

*Section on Surgery.*—Tuesday evening, January 7. Program: Nephrolithiasis, with reports of some interesting cases, Ramon Guiteras, New York City.

*Section on Medicine.*—Tuesday evening, January 14. Program: The diagnosis of gastric cancer, A. L. Benedict; The capillary area, Eli H. Long.

*Ophthalmology, Otology, Laryngology and Rhinology.*—Monday evening, January 20. Program: The eye muscles of animals, with demonstration; tropometers and their uses, Lucien Howe. Report of a case of intraocular hemorrhage occurring during extraction of cataract, Arthur G. Bennett.

*Section on Pathology.*—Tuesday evening, January 21. Program: Relation of evolution to infection with special reference to venereal diseases, G. Frank Lydston, Chicago; discussion by Roswell Park, William H. Heath and Walter D. Greene. New tissue formation, its early detection and complete obliteration, J. Henry Dowd.

*Section on Obstetrics.*—Tuesday evening, January 28. Program: Subject to be announced, William R. Pryor, New York City.

---

THE Medical Union held its 19th annual banquet at the Genesee, Wednesday evening, January 22, 1902, at 7.30 o'clock. The banquet committee was Drs. A. H. Briggs, R. L. Banta, Frederick Preiss. Dr. A. H. Briggs acted as toastmaster. The toasts were: "The Medical Union," J. H. Potter; "Prodigal Son," Conrad Diehl; "Future Health," Health Commissioner Greene; "Tuberculosis," William G. Bissell; "Surgery," Edward J. Meyer; "The Old Practitioner," William C. Phelps; "Fraternity," J. C. Thompson. Among those at the banquet table were Drs. Banta, Hubbell, Renner, A. E. Diehl, Congdon, Dorr,

D. C. Greene, S. S. Green, Kenerson, Rasbach, Hawley, Buswell, McMichael, Fell, Ingraham and Frederick Preiss.

